# Cell-Free Propagation of *Coxiella burnetii* Does Not Affect Its Relative Virulence

**DOI:** 10.1371/journal.pone.0121661

**Published:** 2015-03-20

**Authors:** Runa Kuley, Hilde E. Smith, Dimitrios Frangoulidis, Mari A. Smits, Hendrik I. Jan Roest, Alex Bossers

**Affiliations:** 1 Department of Infection Biology, Central Veterinary Institute part of Wageningen UR, Lelystad, The Netherlands; 2 Department of Bacteriology and TSEs, Central Veterinary Institute part of Wageningen UR, Lelystad, The Netherlands; 3 Host Microbe Interactomics, Wageningen University, Wageningen, The Netherlands; 4 Bundeswehr Institute of Microbiology, Munich, Germany; University of Arkansas for Medical Sciences, UNITED STATES

## Abstract

Q fever is caused by the obligate intracellular bacterium *Coxiella burnetii*. *In vitro* growth of the bacterium is usually limited to viable eukaryotic host cells imposing experimental constraints for molecular studies, such as the identification and characterisation of major virulence factors. Studies of pathogenicity may benefit from the recent development of an extracellular growth medium for *C*. *burnetii*. However, it is crucial to investigate the consistency of the virulence phenotype of strains propagated by the two fundamentally different culturing systems. In the present study, we assessed the viability of *C*. *burnetii* and the lipopolysaccaride (LPS) encoding region of the bacteria in both culture systems as indirect but key parameters to the infection potential of *C*. *burnetii*. Propidium monoazide (PMA) treatment-based real-time PCR was used for enumeration of viable *C*. *burnetii* which were validated by fluorescent infectious focus forming unit counting assays. Furthermore, RNA isolated from *C*. *burnetii*propagated in both the culture systems was examined for LPS-related gene expression. All thus far known LPS-related genes were found to be expressed in early passages in both culturing systems indicating the presence of predominantly the phase I form of *C*. *burnetii*. Finally, we used immune-competent mice to provide direct evidence, that the relative virulence of different *C*. *burnetii* strains is essentially the same for both axenic and cell-based methods of propagation.

## Introduction

*Coxiella burnetii*, the etiological agent of Q fever, is an obligate intracellular bacterium that multiplies within a modified phagolysosome of eukaryotic cells. Q fever is primarily a zoonotic infection that is transmitted via inhalation of contaminated aerosols associated with domestic livestock operations [1]. In humans Q fever manifests as acute and chronic infections. Acute infection symptoms commonly include fever, pneumonia or hepatitis [2, 3] whereas persistent infections lead to chronic disease commonly presenting as endocarditis, which is difficult to treat with antibiotics [4]. A large outbreak occurred in the Netherlands during the years 2007–2010 where more than 40,000 people were assumed to be infected [5–7]. The causes for the outbreak are not fully understood and one of the main reasons speculated were the hyper virulent behaviour of the circulating strains which might have resulted in the increased zoonotic potential [6, 8, 9]. Unfortunately, more accessible cell-free cultivation [10, 11] of *C*. *burnetii* to be used in, for instance genetic modification or sample multiplication studies were not linked to virulence measurements yet. Validation of such tools assists in improved understanding of the pathogenesis of *C*. *burnetii*, which in turn may contribute to a more efficient control of the disease.

The obligate intracellular nature of *C*. *burnetii* imposes several obstacles and makes it to be largely experimentally intractable to use molecular-genetic approaches aiming at a better understanding of its pathogenesis and virulence factors. Studying these factors of *C*. *burnetii* may benefit from the recent development of an extracellular growth medium [10, 11]. However, it is crucial to know whether the viability of *C*. *burnetii* strains, as well as aspects of phase variation and virulence characteristics is not affected by the two different culturing systems. So far procedures to determine the viability of the bacteria based on direct enumeration methods (for instance colony counting) are not easily achievable for *C*. *burnetii*, although this is needed to accurately determine inoculation doses in animal experiments. Less often the viability of bacteria is measured by counting the infectious plaques/colonies in cells infected with serially diluted samples, but this method is tedious, less sensitive and limited to cell-based culture systems [12]. Therefore, quantification of bacteria is usually done by total DNA qPCR methods on selected single-copy genes [13–15]. Nevertheless, qPCR method fail to differentiate between live and dead cells showing the necessity to develop easier methods to quantify live amounts of *C*. *burnetii*.

The production of a structurally and antigenetically unique lipopolysaccharide (LPS) molecule is an important feature in *C*. *burnetii* as LPS is one of the important virulence factors of the organism. The presence or absence of LPS also largely correlates with the phase-variation phenomenon in *C*. *burnetii* [16]. From the two phases, phase I is characterised by the presence of full LPS and is typically the virulent variant whereas phase II has a truncated LPS and usually considered avirulent [17]. All the LPS coding genes are located in a 38 kb region in the *C*. *burnetii* genome and it has been observed that chromosomal deletions in this region result in antigenic phase variation from phase I to phase II [18, 19]. The shift from phase I to phase II occurs usually due to repeated passages of the strains in cultures or embryonated eggs [17, 20], but the molecular mechanisms influencing the LPS modifications still remain unclear.

Propagation of *C*. *burnetii* in cell-based cultures is considered the gold standard for *in vitro* growth. The development of an axenic (cell-free) growth medium for *C*. *burnetii* has greatly facilitated genetic manipulation and the isolation of single colonies on solid medium, which is not possible with cell-based cultivation methods [10, 11, 21–24]. However, the cell-free system is an enhanced artificial environment for *C*. *burnetii* cultivation in comparison to cell-based propagation, which closely mimics *in vivo* infection conditions. Although *C*. *burnetii* grown in cell-free medium exhibited developmental forms characteristic of *in vivo* grown organisms and are infective [11], it is not known whether they have altered virulence characteristics compared to *C*. *burnetii* grown in cell-based culture.

Within the scope of virulence factor characterisation, the aim of the present study was to investigate, whether propagation in two different *in vitro* culturing systems affects the viability, phase variation and relative virulence of *C*. *burnetii* strains. To assess viability of the *C*. *burnetii* strains, we used propidium monoazide (PMA) dye after comparing it with ethidium monoazide (EMA) dye for selective PCR amplification of DNA from viable bacterial cells [25] as well as with fluorescent infectious focus forming units counting (FFU) [12, 26, 27] and counting colony forming units (CFU) [10]. To determine the phase of the strains, expression of LPS coding genes was studied by using microarrays on RNA isolated from low and high passaged *C*. *burnetii* strains. The level of gene expression served as an indicator to predict the functional state of the genes present in the LPS coding region. The gene expression studies were further validated by using raw next generation sequencing reads to assess for any potential deletions of genes involved in LPS synthesis. Ultimately, the relative virulent phenotype of *C*. *burnetii* strains was determined by experimental infection using an immune-competent mice model [28–31]. This study provides evidence that cell-free propagation of *C*. *burnetii* does not affect its viability, phase variation and relative virulence as seen by mouse virulence bioassay.

## Materials and Methods

### Animals

Two independent animal experiments were conducted using Specific-Pathogen-Free Swiss female OF1 mice (Charles River, l’Arbresle, France). For Experiment 1 (E1) and Experiment 2 (E2), 6 and 7 week old mice were used respectively. The mice were housed under sterile conditions in biosafety level 3 facilities and acclimatized for one week before experimental infection. Each group was housed separately and consisted of 10 mice.

### Ethics statement

Animal experiments were approved by the animal experiment commission of the Central Veterinary Institute of Wageningen UR, and conducted in accordance with the Dutch regulations on animal experimentation (Registration numbers: 2010011.b, 2012126.a). Humane end points were defined prior to the experiments and all possible measures were taken to minimise animal suffering.

### *C*. *burnetii* strains and cultures

In this study the isolates X09003262–001 (3262); X08014160–001 (601) and X08014160–002 (602) were used. These isolates were primarily isolated from the placentas of goats that aborted during the Q fever outbreak in the Netherlands (2007–2010) [5]. These 3 isolates were molecular typed as 2 genetically different strains (602 and 3262 similar molecular MLVA genotype) [5] and were initially propagated in Buffalo Green Monkey (BGM) cells (European Collection of Cell Cultures, Salisbury, U.K) without antibiotics as described previously [30]. Two additional strains included in the study were Nine-Mile RSA 493 (NM) and Scurry Q217 (scurry) provided by D. Frangoulidis (Bundeswehr Institute of Microbiology, Munich) and were initially propagated in the same way. The stock cultures of all the strains propagated in BGM cells were stored at -80°C. Prior to experimentally infecting mice, the strains were propagated for a few passages (n<4x times) in cell based and cell free culture systems in order to adapt the strains to the new (cell free) culture system [10] and to synchronise the passage numbers of the strains.

### Viability PCR and quantification of *C*. *burnetii*

To determine viability of *C*. *burnetii*, PMA and EMA dyes were compared on strains cultured from cell-based and cell-free culture system. The protocol is essentially described before [25], where EMA was used to a final concentration of 100 μM to assess the viability of *C*. *burnetii*. A similar final concentration of 100 μM of EMA and PMA (Geniul, Spain) was used in this study. Briefly, the dyes were added to the samples in opaque tubes and incubated in dark for 30 min at 4°C and vortexed regularly. The samples were transferred to high transparent tubes and were photo-activated for 30 minutes using PhAST blue system (Genuil, Spain). DNA was isolated from the samples by using NucliSENS easyMag machine (BioMérieux, USA) as per manufacture instructions. One tenth of the DNA was used as a template for detection and quantification of number of *C*. *burnetii* by using a TaqMan-based real-time PCR targeting the single copy gene (CBU_0407a, gene bank number AY502846) encoding a *C*. *burnetii* specific hypothetical protein as previously published [30]. A standard curve was generated by using ADIAVET COX Positive control (Adiagene, Marcy l’Étoile, France) according to the manufacturer’s instructions.

### Fluorescent infectious focus forming unit’s measurements

Viability PCRs were validated by a direct enumeration method using microscopic counting of fluorescent infectious focus forming units (FFU). The viable number of bacterial cells were established by inoculating serial dilutions of bacterial cells (10^–1^ to 10^–9^) from both culture systems on confluent monolayers of BGM cells in 24 well tissue culture plates (Costar, Corning, USA) containing 15 mm coverslips (Menzel GmbH, Germany). After 24 hours incubation at 37°C in 5% CO_2_, infected cells were washed twice and incubated for 6 days. Infected cells were demonstrated by indirect immunofluorescence assay using *C*. *burnetii* specific monoclonal antibody (MAB313-oregon green, Squarix). Number of viable bacteria in the inoculum was determined from the last dilution which resulted in at least one infected cell [12]. To further confirm the FFU counts, CFU counts were also performed with the same bacterial dilutions used for FFU on solid agar plates as mentioned previously [10].

### RNA preparation and microarray analysis

Total RNA was isolated from *C*. *burnetii* strains by using Direct-zol RNA Miniprep Kit (Zymo Research, Irvine, USA) as per manufacturer’s instructions. Poly-A tails were tagged to bacterial total RNA (500 ng) by using Poly-A Polymerase Tailing kit (Epicenter Illumina company, Madison, USA). This RNA was used as starting material for Agilent’s protocols for amplification, labelling, hybridization, washing and scanning of the *C*. *burnetii* microarrays. The *C*. *burnetii* custom microarray consisted of user-designed probes (https://earray.chem.agilent.com/earray) covering the complete LPS-gene repertoire of several *C*. *burnetii* strains (NCBI, Accession numbers: AE016828.2, CP000733.1, CP001020.1, CP001019.1, CP001021.1) and were synthesized by Agilent Technologies (Santa Clara, CA, USA). Data was obtained through Agilent's feature extraction software (Version 10.7.3.1) and analysed by using GeneSpring software (Agilent Technologies).

### Genome coverage analysis

Total DNA from high passage *C*. *burnetii* strains (n>30x times) propagated in cell-free culture was isolated by phenol-chloroform method [32] after overnight incubation with ATL lysis buffer and proteinase K (QIAGEN, Hilden, Germany). Whole genome sequencing was performed using 250 bp paired-end sequencing libraries (Nextera TAG-mentation sequencing kits, Epicentre, Madison, USA) on an Illumina MiSeq sequencer. High quality paired end reads were mapped against the reference NM RSA493 genome available from the NCBI database (Accession number: AE016828.2) using Bowtie2 alignment tool [33]. The average coverage and standard deviation (excluding repetitive regions) were obtained for the whole genome using a sliding-window approach and were used to calculate the coverage of genes involved in LPS synthesis (CBU0676 to CBU0706). A coverage below the minus 2 SD interval was considered as a significantly deleted gene. Apart from genome analysis, the protein profiles of low and high passaged 602 strain were also analysed by SDS-PAGE using the whole cell lysates, as described previously [34]. The method was basically the same except for staining of proteins with SYPRO Orange dye (Molecular probes) and visualising the protein profiles with a Typhoon Trio imager and ImageQuant 5.2 software (Molecular Dynamics).

### Infection mice and collection of tissues

Two independent infection experiments were performed where 10 Swiss OF1 mice per strain were inoculated intraperitoneally with 0.2 ml suspension of *C*. *burnetii* in phosphate buffer saline (PBS) in order that each mouse receives a dose of 10^5^ genome copy equivalents as determined by quantitative PCR. PBS was used for the negative controls. The inocula used to infect mice were tested for number of viable cells after the infection experiments by using PMA-PCR. The samples were freeze thawed once and similar percentages (55–65%) of alive bacterial cells were found in the inocula. All the strains from both culture systems were found to be in phase I as measured by expression profiles of LPS encoding genes using a microarray. One week after inoculation the mice were euthanized, weighed and their spleen were aseptically removed, weighed and frozen at -80°C. Weights were used for determining relative spleen weights (means of spleen weight/body weight (%)).

### Measurements of bacterial loads from spleens of mice

To quantify the number of *C*. *burnetii* in mice spleens, DNA was extracted from the spleen by using the DNeasy Blood and Tissue Kit (QIAGEN, Hilden, Germany). Briefly, the spleen was homogenised after addition of PBS. About 200 μl suspension was taken and added to tissue lysis buffer (buffer ATL) and proteinase K. DNA was extracted from this suspension according to the protocol for gram-negative bacteria as recommended by the manufacturer.

The DNA was used as a template for detection and quantification of number of *C*. *burnetii* by using a TaqMan-based real-time PCR as explained above. The bacterial genome copy number from PCR was log transformed and determined as the mean log2 values per gram spleen.

### Statistical analysis

Samples and sample-derived data from each mouse were kept separate for all treatments and analysis. Data from viability of PMA treated samples, normalised expression of LPS genes and relative virulence from groups of mice are expressed as means ± standard deviation and compared by independent two tailed student *t*-tests. Differences were considered significant at a *p* value of <0.01.

## Results

### Evaluation and comparison of PMA and EMA dyes for the *C*. *burnetii* live/dead assay

**Fig 1:**
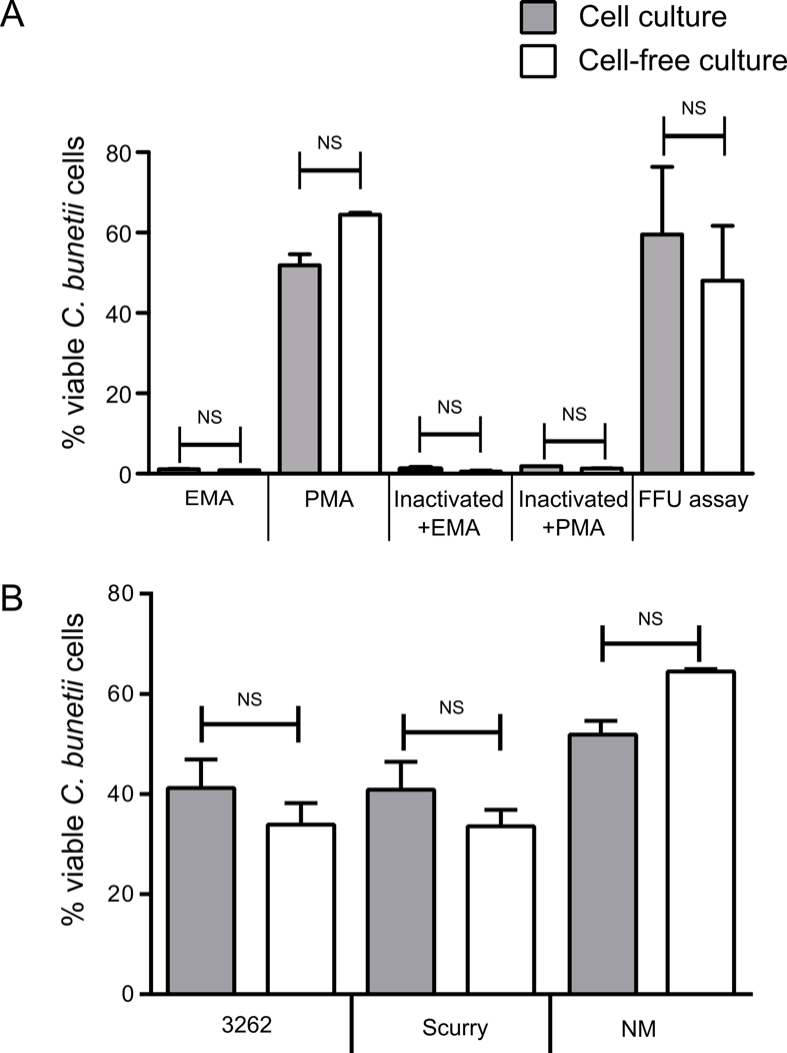
Viability PCR of *C*. *burnetii* on the single copy gene in cell-based and cell-free matrix. A) Comparison of PMA and EMA PCR with limited dilution FFU assays. PMA and EMA measurements were performed on normal and heat-inactivated (dead) cells of *C*. *burnetii*. FFU assays were performed to validate PMA-PCR measurements. B) *C*. *burnetii* strains were exposed with PMA and the genomic DNA yield was expressed as a percentage of alive cells corresponding to non-PMA-treated sample. The error bars represent standard deviations from two independent replicates. NS indicates *p*-value > 0.01.

Propidium monoazide (PMA) and Ethidium monoazide (EMA) were utilized to selectively allow real-time PCR amplification of target DNA from viable *C*. *burnetii* cells. The basic principle involves selective penetration of these dyes only into dead bacterial cells with compromised membrane integrity but not into the live cells with intact membranes. Once inside the cells, the dyes cross link with DNA upon exposure to bright light and inhibits its amplification by PCR [35]. In the present study serially diluted *C*. *burnetii* (NM strain) containing approximately 10^6^ cells/ml from cell-based and cell-free culture matrix were treated with EMA and PMA. The results show a clear difference between the two dyes. In PMA treated samples 50–60% of alive cells were detected, whereas in EMA treated samples only few cells (1–2%) were detected to be viable based on the DNA amplification compared to untreated samples (Fig. 1A). We concluded that, PMA dye selectively entered dead cells that allowed efficient amplification of live *C*. *burnetii*, whereas EMA inhibits DNA amplification of almost all cells. To validate the efficiency of PMA-PCR, limiting dilution FFU assays were conducted to measure the number of viable cells based on the last dilution that resulted in at least one infected cell. Similar amounts of viable bacteria from both the culture systems were measured as seen by PMA-PCR (Fig. 1A). Furthermore, CFU counts [10] also confirmed the FFU counts, where the last dilution resulting in colonies corresponded with the same dilution which resulted in at least one infected cell in FFU assay (data not shown). To test the potential in discriminating live and dead cells by these dyes, the samples were subjected to heat treatment at 99°C for 30 min. Very high signal reduction was seen in samples both treated with EMA and PMA with >99% decrease in DNA amplification. To further test the reproducibility of this technique, different strains from cell-based and cell-free culture (3262, Scurry and NM) were tested with PMA followed by qPCR. In PMA treated samples 30–65% of alive cells were detected based on the DNA amplification (Fig. 1B) with respect to non-PMA treated samples. No significant differences were seen between culture systems with respect to viability of cells (Fig. 1B).

### Gene expression in LPS coding genes of *C*. *burnetii*

**Fig 2:**
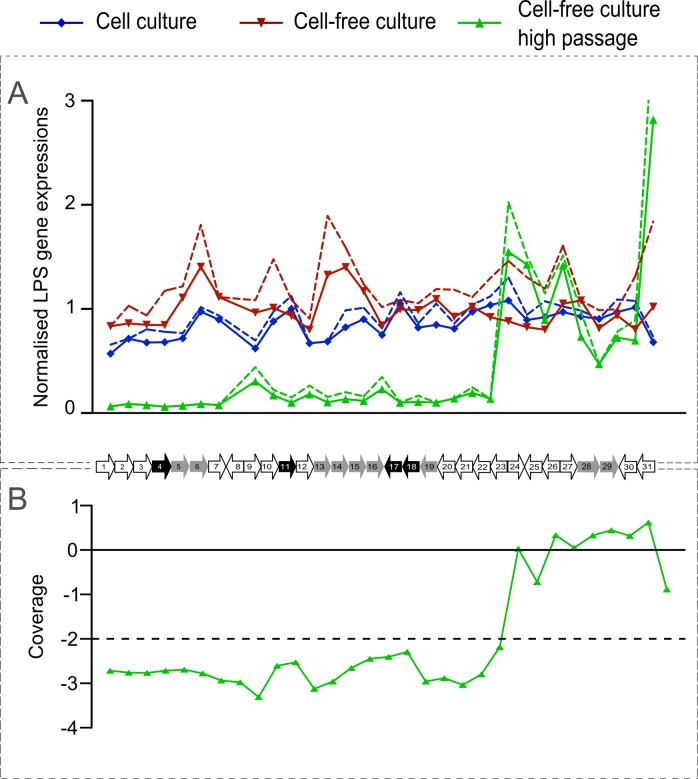
Normalised LPS gene expression profiles and genome coverage of *C*. *burnetii* 602 strain as a representative example. A) Here average transcript signal per gene was normalised with the average transcript signal of housekeeping gene *rpoB* of *C*. *burnetii*. The normalised gene expressions are plotted for 602 low passage strain in cell-based and cell-free culture systems and 602 high passaged cell-free culture strain. The dashed lines represent standard deviations from two independent replicates. B) Genomic coverage of genes involved in LPS synthesis of a high passaged cell-free culture 602 strain. A coverage below the minus 2 SD interval (dashed line) was considered as a significantly deleted gene. The x-axis for both the A and B graphs are shown as a scaled mini-map of *C*. *burnetii* LPS encoding genes. Each data point represents to its corresponding gene (CBU0676 to CBU0706) labelled as 1 to 31 genes in the gene map. The genes indicated in grey are hypothesized to be involved O-antigen synthesis and export. Genes indicated in black are hypothesized to be involved in other LPS synthesis steps and carbohydrate metabolism.

Within the 38 kb region of *C*. *burnetii* strains the junctions of large, nested deletions are located which can lead to phase variation [18]. We first tried to find evidence for the presence or absence of these deletions using a PCR approach (data not shown) targeting on multiple locations in this region. By this approach, we did not find any clear evidence for the presence of deletions as would be seen in a phase II variant [18]. To obtain additional evidence for the absence of LPS gene deletions, we subsequently used a microarray approach to measure the expression level of genes in this region in cells grown in both cell-based and cell-free culture systems (Fig. 2A). The average gene expression was normalised with expression of the housekeeping gene *rpoB* [36] of *C*. *burnetii* and was plotted for the 602 strain as a representative example. Here, a measurable expression indicates the presence of transcribed RNA from genes located in the 38 kb region, while no expression points towards a possible loss of function due to regulation or deletion events. The comparison of normalised LPS gene expressions from strain 602 shows similar expression levels for all genes in the LPS encoding region in both cell-based and cell-free cultured strains. This supports the earlier conclusion that for both culturing systems no deletion in the LPS region could be detected. A similar trend of LPS expression is seen in other strains (data not shown) that were used in the virulence bioassay of mice as described in this study, indicating that the majority of cells as cultured by low passage-number in the cell-based and cell-free system are in phase I. In contrast, high passaged 602 strain from cell-free culture showed a very low expression of LPS genes at the beginning of the LPS encoding region (CBU0676 to CBU0697) and high expression of the genes towards the end (CBU0698 to CBU0706) (Fig. 2A). Comparing the regulation of LPS encoding genes of high passaged cell-free culture with low passaged cell-based and cell-free culture strains showed a significant (p<0.01) down regulation of CBU0676 to CBU0697 genes with a minimum fold change < 4 (data not shown). These significant difference between the higher passaged cells (n>30x times) and the lower passaged cells (n<10x times) shows loss of function of the genes (CBU0676 to CBU0697) at the beginning of the LPS cluster and a consecutive variation from phase I to phase II in a significant proportion of the cell population.

### Deletion of genes involved in LPS formation by whole genome sequence analysis

The low expression of genes involved in LPS synthesis in the high passaged *C*. *burnetii* strain were further analysed for any deletions by whole genome sequencing. The coverage of reads for LPS encoding genes (CBU0676 to CBU0697) were significantly lower than the genes present at the end (CBU0698 to CBU0706) of the LPS encoding region (Fig. 2B) indicating deletions of these genes in a significant proportion of the cell population and a phase variation moving into a phase II form. The data also demonstrated that the occurrence of (growing) deletions is most likely an on-going event during passage since the read-mapping indicates a mixed metagenomic population at this LPS encoding region of the genome.

### Mouse bioassay

All mice remained clinically healthy during the course of the experiment. However, at autopsy mice inoculated with *C*. *burnetii* strains showed significant splenomegaly as compared to control mice. The mice bioassay’s showed a statistically significant level of splenomegaly in infected animals compared to the respective controls (*p*<0.01), except for the cell-based grown Scurry strain (E1) group (*p* = 0.10).

### Relative virulence of *C*. *burnetii* strains in mice

**Fig 3:**
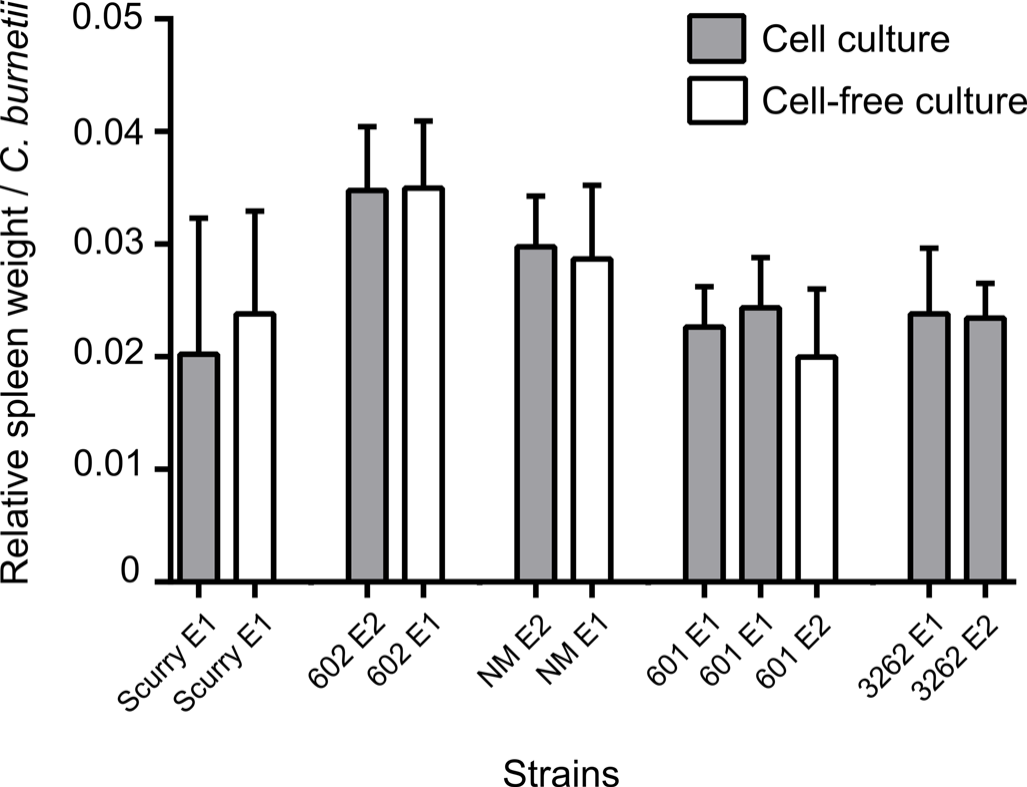
Changes in relative virulence. Relative virulence of different strains cultured in cell-based and cell-free systems expressed as the relative spleen weight per number of *C*. *burnetii*. E1, experiment 1; E2, experiment 2.

The relative virulence of *C*. *burnetii* strains was expressed as the “relative spleen weight per number of *C*. *burnetii* bacteria” which considers both the degree of splenomegaly and the number of *C*. *burnetii* present in the spleen of infected mice. The *in vivo* growth of bacteria in the mice spleens were measured by qPCR assay, where the bacterial DNA was quantified by CBU_0407a single copy gene (results not shown). As shown in Fig. 3 and Table 1, the relative virulence of a particular strain is not significantly affected by the *in vitro* system that is used to propagate the bacteria (*p*>0.01). These results indicate that the phenotypic expressions of virulence of the used *C*. *burnetii* strains in mice is independent of the *in vitro* culture system chosen for propagating the strains. No statistically significant differences for the relative virulence (*p*>0.01) were obtained between cell-based cultured strains of 3262 and 601 in these two independent experiments (E1 and E2), clearly highlighting the reproducibility for this measure in the mouse virulence bioassay.

**Table 1:** A significant difference matrix of *C*. *burnetii* strains from cell or cell-free culture evaluated for relative virulence in a mouse bioassay.

Strain	Source	Country	Culture	Scurry		NM		602		601			3262	
				cell^a^	cf^b^	cell	cf	cell	cf	cell E1^c^	cell E2^d^	cf	cell E1	cell E2
Scurry	human	USA	cell	n.a										
			cf	-	n.a									
NM	tick	USA	cell	-	-	n.a								
			cf	-	-	-	n.a							
602	goat	NL	cell	*	*	-	-	n.a						
			cf	*	*	-	-	-	n.a					
601	goat	NL	cell E1	-	-	*	-	*	*	n.a				
			cell E2	-	-	-	-	*	*	-	n.a			
			cf	-	-	*	*	*	*	-	-	n.a		
3262	goat	NL	cell E1	-	-	-	-	*	*	-	-	-	n.a	
			cell E2	-	-	*	-	*	*	-	-	-	-	n.a

Significant differences in relative virulence between different strains (where *p*<0.01, * and *p*>0.01,-).

- non-significant differences between cell and cell-free cultures.

n.a, Indicate non applicable insignificant self-comparisons.

^a^ Cell, Cell-culture;

^b^ cf, Cell-free culture;

^c^ E1, experiment 1;

^d^ E2, experiment 2.

## Discussion

In the present study we used three different methods to evaluate virulence-associated characteristics of different *C*. *burnetii* strains cultured in cell-based and cell-free systems. We did not observe any difference in the number of viable cells in the two culturing systems as determined by the use of PMA-PCR. Furthermore, we did not find any evidence for the occurrence of loss of function of genes in the LPS encoding region in the low passaged strains from both the culturing systems. Since the loss of gene function of the genes in the LPS cluster can be associated with the variation of the virulent phase I to the avirulent phase II, we do not expect significant differences in the phase I to phase II ratios in the low passages of *C*. *burnetii* strains in both the culturing systems. Finally, we provide evidence that the relative virulence of *C*. *burnetii* strains is not affected by the cell-based or cell-free method of propagation as measured in an immune-competent mouse model. Therefore, our results clearly demonstrate that the cell-free culture system does not significantly influence the relative virulent phenotype of the tested strains. This indicates that the cell-free culturing system is invaluable for the identification and characterisation of virulence factors of *C*. *burnetii* while allowing the investigation of molecular mechanisms involved in the pathogenesis of this obligate intracellular bacterium.

Our study reports the use of PMA-PCR, which worked better in our hands compared with EMA-PCR for quantification of viable cells of *C*. *burnetii*. The efficient quantification of genomic DNA from live cells by PMA or EMA treatment followed by PCR has been reported previously in different bacterial species [25, 35, 37–39]. It is one of the few current methods available for *C*. *burnetii* [25], which can be used to determine and quantify viable cells. Compared to PMA-PCR, decrease in PCR amplification was seen in EMA-PCR resulting in very low numbers of viable cells. In previous studies, EMA treatment showed loss of more than 60% genomic DNA due to penetration in live cells, whereas PMA was found to selectively penetrate only dead cells [35, 37, 39, 40]. This higher impermeability of PMA through the intact cells might be due to the higher charge of PMA as suggested previously [35]. In samples treated at high temperatures, a high decrease in PCR amplification efficiency was seen compared to untreated cells, consistent with previously observations in other bacterial species. Therefore, a complete inhibition of PCR amplification of DNA from dead cells could not be expected at the tested concentrations of *C*. *burnetii* cells [39, 41, 42]. One of the important parameters to consider in this method is the matrix of the culture system the bacteria were propagated in, as non-specific host DNA also contributes in the efficiency of this technique. EMA-PCR seemed to work well in egg homogenate matrix of *C*. *burnetii* which contains very high amounts of host DNA as previously described [25], but not on cell-based culture matrix which contain relatively less host DNA or no host DNA as in cell-free culture matrix. Hence, PMA-PCR was seen to mitigate the drawbacks observed with EMA-PCR in cell-based and cell-free culture systems. Also the viability counts obtained by PMA-PCR corresponded closely with direct enumeration FFU assay and CFU assay (data not shown) showing it as an efficient method to enumerate viable *C*. *burnetii*.

Transcriptomics studies of all LPS encoding genes located in a 38 kb region in the *C*. *burnetii* genome is one of the robust and easy ways to detect loss of function of all these genes, which basically results in phase variation. The measured gene expression profiles showed all the low passaged strains of *C*. *burnetii* to be in phase I. Several other molecular techniques can also be used to determine the *C*. *burnetii* phase variation including approaches such as determining LPS sugar composition and quantification [43–46], PCR’s on all genes of LPS coding regions [19], microarray-based genome comparisons [47], reverse transcriptase-PCR for expression of genes [19] and genome sequencing [18, 19]. These methods are rather elaborative and tedious. Our result of transcriptomic measurements of a representative low passaged strain shows similar levels of expression of all genes in the LPS encoding region, without any significant differences in both cell-based and cell-free culture system. However, the high passaged cell-free cultured strain, showed significant (p<0.01) lower expression of several genes (CBU0676 to CBU0697) in major part of this region, compared with the low passaged cell-based and cell-free culture systems. Further, significant (p<0.01) down regulation of these genes with a fold change of < 4 confirms the possible loss of gene function, which can result in a transition from phase I to phase II phenotype of the bacterium. This significant decrease of transcripts could also be regulatory and/or a physical deletion event within the LPS genes. Although lower, the expression of these genes are still detected suggesting the presence of mixtures of phase I and II variants of the strain, where the observed remaining transcripts of these genes are most likely from bacteria still in phase I form. High expression of genes were seen in this strain towards the end of the region (CBU0698 to CBU0706) suggesting the possible remains of these regions in the phase II variants.

**Fig 4:**
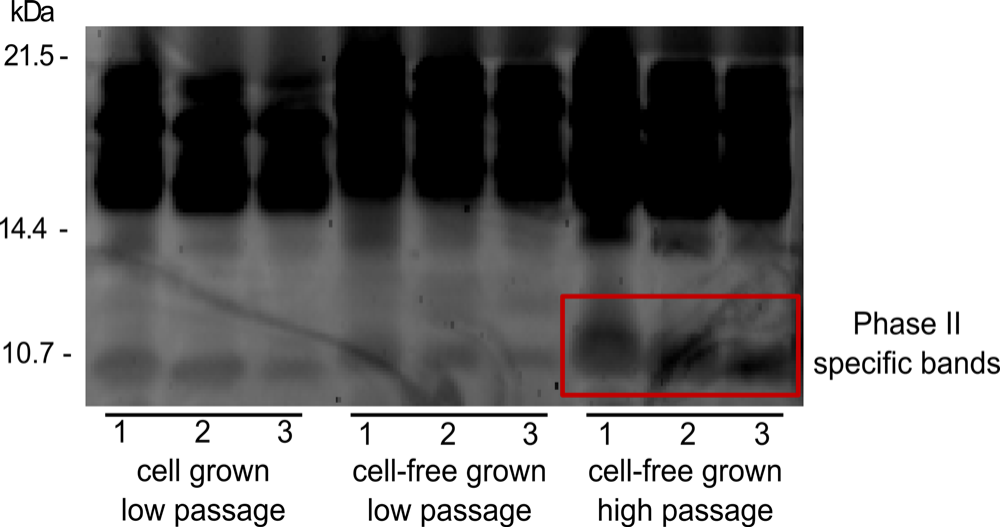
SDS-PAGE of *C*. *burnetii* whole cell lysates of low passaged cell-based and cell-free cultures and high passaged cell-free cultured 602 strain. Three serial dilutions (1:5) marked as 1, 2 and 3 for all samples were used. The red box indicates the phase II specific bands seen in high passaged cell-free cultured strain.

Whole genome sequencing was performed to investigate whether any deletion events result in the observed low expression/regulation of genes involved in LPS synthesis. Here we used the depth of coverage in whole genome sequence data. A significant decrease in coverage was observed in the genes which showed low expression (CBU0676 to CBU0697) indicative for a deletion. Some of the deleted genes are involved in O-antigen biosynthesis or other LPS biosynthetic steps (Fig. 2) and its deletion may result in severe impairment of LPS synthesis correlating with phase variation [18, 48]. Such large deletions were also seen previously in other phase II strains such as 9Mi/Baca (deletion from CBU0678 to CBU0694), 9Mi/II/C1 and 9Mi/II/C4 (deletion from CBU0678 to CBU0698) [18, 19]. The coverage of the genes (CBU0698 to CBU0706) present at the end of the LPS encoding region were higher, corresponding to the observed normal expression of these genes. Hence, the lower and higher read depths of genes obtained from sequencing (Fig. 2B) corresponded to lower and higher expression of the same genes obtained from expression studies (Fig. 2A). Similar results were also seen from SDS-PAGE analysis, where whole cell lysates of low and high passaged 602 strain had a similar profile of protein fragments which reflected those of the phase 1 type (bands between 21–14 kDa). In addition, the high passaged strain comparatively had a more prominent band at 10.7 kDa which is characteristic of phase II type. Hence, both phase I and phase II characteristic profiles were observed in high passaged cell-free cultured strains (Fig. 4) [34]. We conclude that low passaged strains from both the culture systems were in a predominant phase I form, and high passaged cell-free cultured strain shows possible deletions resulting in loss of gene function in the beginning of this LPS encoding region which is progressing throughout the region resulting in a phase II variant of the strain. Hence, cell surface LPS of *C*. *burnetii* is not significantly changed in the cell-free culture system similar to cell-based culture system in its early passages. Eventually phase variation occur due to multiple passages in cell-free culture system, due to loss of LPS side chain as suggested previously [49].

In this present study we used immune-competent Swiss OF1 mice to evaluate the virulence properties of different *C*. *burnetii* strains. This model is currently used by several groups for evaluating pathogenicity of *C*. *burnetii* with consistent outcomes [28]. We assessed the virulence of *C*. *burnetii* strains using “splenomegaly” (increase in weight of spleen in relation to the body weight) and “RT-PCR quantification of bacteria in the spleen” as the most important read-out parameters [13, 15, 50]. Splenomegaly in response to infection was observed in these mice without any adverse clinical signs or mortality at 7 days post infection. Even at 21 days after infection no mortality of mice was observed and the splenomegaly reduced as well as the number of bacteria in the spleens, most likely indicating a level of clearance of the infection (data not shown). Also the degree of splenomegaly correlated with the bacterial load in the spleen as explained before [51]. All strains were capable of inducing pathological changes in the Swiss OF1 mice, showing its sensitivity to Q fever agent.

The strains propagated in cell-based and cell-free culture systems did not show any significant differences in their relative virulence, showing relative virulence is independent of the culture system in which the strains are propagated in. We combined both, splenomegaly and bacterial load, which are most likely related, as virulence parameters to determine relative virulence of *C*. *burnetii*. The extent of splenomegaly and presence of *C*. *burnetii* in the spleen was strain dependent indicating that the strains most likely differentially affect the influx of (T) cells into the spleen and thereby facilitate bacterial clearance [15]. The colonization of spleen was a good indicator of infectivity, highlighting the relevance of these measurements for comparing strain virulence. Hence, the relative virulence showed strain-specific (*p*<0.01) but similar virulence values (*p*>0.01) for the same strain, irrespective the method of *in vitro* propagation for the tested low passage strains (Table 1). It is also expected that strains propagated from both the culture systems would equally loose virulence with respect to higher passages due to loss of LPS structure as seen previously [49]. Finally, the two individual experiments conducted showed reproducible relative virulence in 3262 and 601 cell-based cultured strains, underlining the precision of virulence associated measurements in the present mouse virulence bioassay.

In conclusion, our results provide evidence that the axenic culture system does not significantly influence the viability, phase variation and relative virulence of *C*. *burnetii* strains compared with cell-based culture. Thereby the cell-free propagation is an invaluable tool when studying molecular mechanisms underlying differences in virulence by using molecular genetic studies, which are not feasible using cell-based cultivation. Ultimately this system could be useful for development of effective subunit vaccines or production of recombinant antigens, which would offer great potential in the control of Q fever outbreaks as well as limiting its transmission to humans.
